# Valve-Specific Anatomy and Structural Determinants of Susceptibility to Infective Endocarditis: A Review

**DOI:** 10.3390/jcm15166281

**Published:** 2026-08-13

**Authors:** Muhd Najmi Hakim Abd Rani, Afifah Mohamed, Zaleha Md Isa, Suhaini Kadiman, Taty Anna Kamarudin

**Affiliations:** 1 Department of Anatomy, Faculty of Medicine, Universiti Kebangsaan Malaysia, Kuala Lumpur 56000, Malaysia; 2 Non-Invasive Cardiovascular Laboratory, National Heart Institute, Kuala Lumpur 50400, Malaysia; najmi@ijn.com.my; 3 Centre for Diagnostic, Therapeutic & Investigative Studies (CODTIS), Faculty of Health Sciences, Universiti Kebangsaan Malaysia, Kuala Lumpur 50300, Malaysia; afifahmohamed@ukm.edu.my; 4 Department of Public Health Medicine, Faculty of Medicine, Universiti Kebangsaan Malaysia, Kuala Lumpur 56000, Malaysia; zms@hctm.ukm.edu.my; 5 Department of Anesthesiology and Critical Care, National Heart Institute, Kuala Lumpur 50400, Malaysia; suhaini@ijn.com.my

**Keywords:** infective endocarditis, valve anatomy, hemodynamics, bicuspid aortic valve, prosthetic valve endocarditis, TAVR, mitral valve prolapse

## Abstract

**Background/Objectives:** Infective endocarditis (IE) is a life-threatening cardiovascular infection with in-hospital mortality of 15–30% despite modern therapy. Contemporary IE demonstrates non-random valve involvement: aortic and mitral 35–45%, tricuspid 5–10% (30–50% in intravenous drug users [IVDU]), and pulmonary < 1%. These patterns implicate valve-specific anatomy and hemodynamics as central determinants of susceptibility. This narrative review examines the reported distribution of IE across the aortic, mitral, tricuspid and pulmonary valves and summarises the anatomical, haemodynamic, structural, microbial and patient-related factors associated with valve-specific susceptibility. **Methods:** A structured narrative review of English-language literature was conducted using PubMed/MEDLINE, Embase, Scopus, and Google Scholar (January 1990–March 2026). Search terms included “infective endocarditis,” “valve anatomy,” “hemodynamics,” “bicuspid aortic valve,” “prosthetic valve endocarditis,” and “transcatheter aortic valve replacement (TAVR) endocarditis.” We included anatomical studies, clinical cohorts, surgical series, imaging research, and international guidelines. Evidence was synthesized narratively using Oxford Centre for Evidence-Based Medicine (CEBM) levels. **Results:** IE susceptibility follows a biologically coherent gradient determined by the interaction between valve anatomy, hemodynamic stress, endothelial injury, and structural substrate. The aortic valve is most vulnerable because of high shear stress, congenital abnormalities such as bicuspid aortic valve, and direct continuity with the cardiac fibrous skeleton, predisposing to peri-annular extension. Mitral valve IE is largely conditional upon pre-existing structural disease, particularly mitral valve prolapse, rheumatic heart disease, and mitral annular calcification, and is characterized by a high risk of systemic embolization. Tricuspid valve IE reflects the interaction between low-pressure hemodynamics and acquired patient-specific modifiers, including intravenous drug use, cardiovascular implantable electronic devices, and congenital heart disease. Pulmonary valve IE remains uncommon because of favorable native hemodynamics but occurs predominantly in repaired congenital heart disease, right ventricular outflow tract reconstruction, and prosthetic pulmonary valves. Across all valve types, multimodality imaging and anatomical assessment consistently influence complication detection, surgical planning, and long-term surveillance. **Conclusions:** IE involvement is unevenly distributed among the cardiac valves. Aortic and mitral involvement predominate, tricuspid involvement is strongly influenced by injection drug use and intracardiac devices, and pulmonary-valve IE remains rare and is principally associated with congenital abnormalities or prosthetic material. These patterns highlight the possible contributions of haemodynamic stress, pre-existing structural abnormalities, and age-related valvular changes to the greater susceptibility of left-sided valves.

## 1. Introduction

Infective endocarditis (IE) remains one of the most devastating cardiovascular infections, with reported in-hospital mortality of 15–30% and one-year mortality approaching 40% despite substantial advances in antimicrobial therapy, cardiac imaging, and surgical intervention [1,2]. The global incidence continues to increase, currently ranging from 3–10 cases per 100,000 population annually, driven by ageing populations, the expanding use of prosthetic valves and cardiovascular implantable electronic devices, healthcare-associated bloodstream infections, and the growing burden of intravenous drug use (IVDU) [3,4].

Despite these evolving epidemiological trends, the distribution of valve involvement has remained remarkably consistent over several decades. The aortic valve accounts for approximately 35–45% of IE cases, with the mitral valve accounting for a similar proportion (35–45%), tricuspid valve (5–10%; up to 30–50% among people who inject drugs), and pulmonary valve (<1%) [5,6]. This reproducible pattern persists despite the transition from rheumatic heart disease to degenerative valvular disease, prosthetic valve implantation, and healthcare-associated infection as the predominant predisposing conditions [7,8]. Such observations strongly suggest that valve-specific anatomical structure and local hemodynamic characteristics, rather than chance alone, are fundamental determinants of IE susceptibility [9].

Although numerous reviews have comprehensively examined the clinical presentation, microbiology, diagnostic imaging, and therapeutic management of infective endocarditis [10,11,12,13,14,15,16,17,18,19,20,21,22,23], comparatively little attention has been directed toward the anatomical and biomechanical factors that determine why individual cardiac valves differ markedly in their susceptibility to infection [12,13,24]. Existing literature generally considers IE as a single clinicopathological entity, with limited integration of how normal valve anatomy, congenital and acquired structural abnormalities, local hemodynamic environments, and pathological remodeling collectively influence disease initiation, progression, and valve-specific clinical behavior [12,13,24].

A clearer understanding of these anatomical determinants has important clinical implications because it may improve risk stratification, surveillance of high-risk patients, selection of imaging modalities, timing of surgical intervention, and future preventive strategies [10,11,14,15,16,17,18,19,20,21,22,23,25,26].

## 2. Methods

This structured narrative review used systematic literature identification of English-language publications indexed in PubMed/MEDLINE, Embase, Scopus, and Google Scholar from January 1990 to March 2026. A total of 1295 records were identified, comprising 1250 database records and 45 additional records. After duplicate removal, title and abstract screening, and full-text eligibility assessment, 85 sources were included in the narrative synthesis. Evidence was synthesized narratively, with the study-selection process shown in Figure S1.

Search terms were developed iteratively and combined using Boolean operators. Core concepts included “infective endocarditis” in conjunction with valve- and mechanism-specific terms such as “valve susceptibility,” “aortic valve endocarditis,” “mitral valve endocarditis,” “tricuspid valve endocarditis,” “perivalvular extension,” and “cardiac abscess.” Reference lists of relevant articles were manually screened to identify additional influential studies not captured by electronic search.

**Figure 1:**
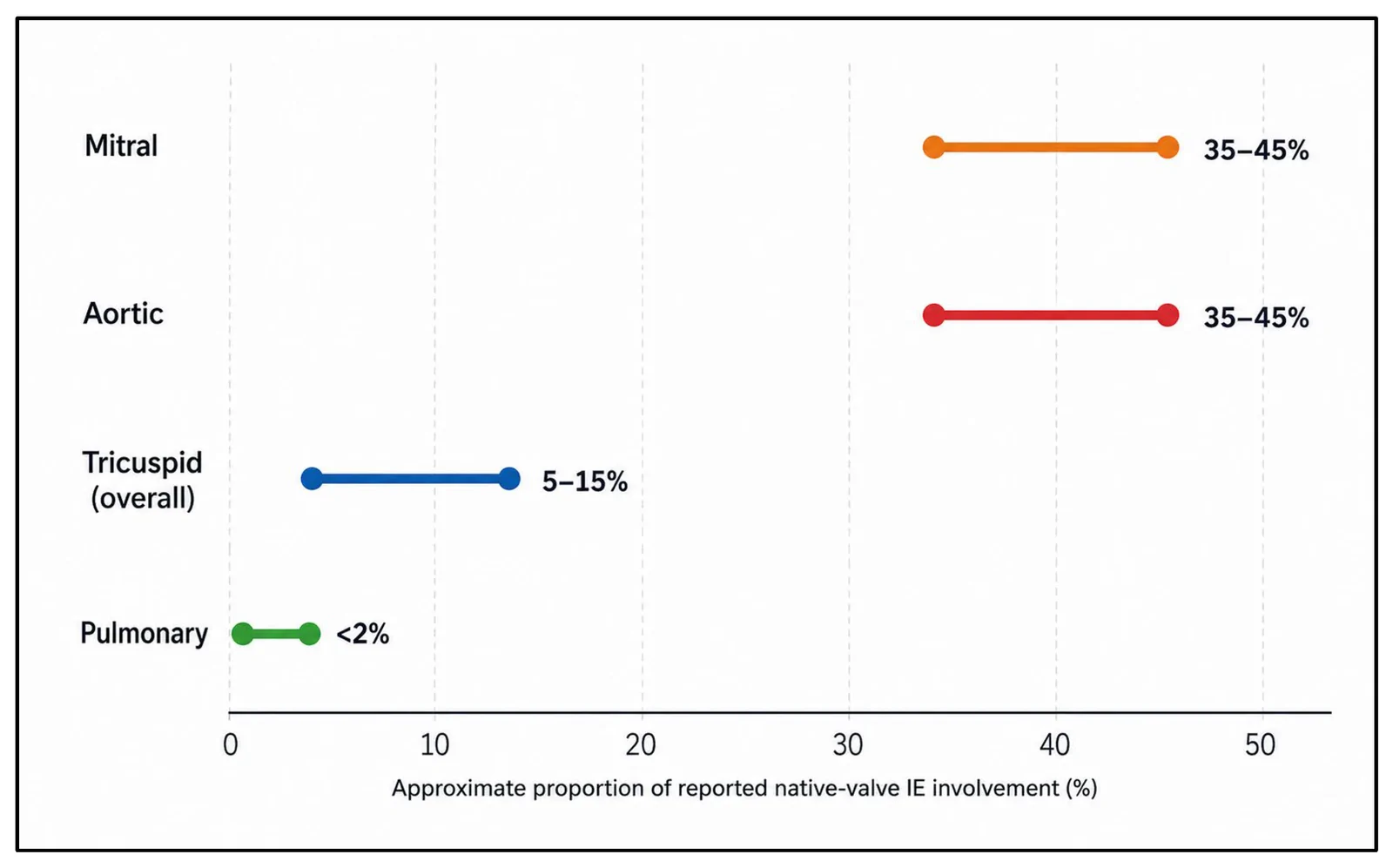
**Distribution of native-valve infective endocarditis.** Approximate proportions of native-valve infective endocarditis involving the mitral, aortic, tricuspid and pulmonary valves, synthesized from published observational cohorts and epidemiological reviews.

Eligible sources included observational cohort studies, registry-based analyses, imaging studies, narrative and systematic reviews, international clinical practice guidelines, and cardiovascular consensus statements published in English. For the principal population-level estimates underlying Figure 1, priority was given to observational cohorts or registries reporting a clearly defined observation period of at least 1 year but fewer than 10 years and including approximately 500 or more patients. Smaller cohorts, reviews, and studies enriched for specific populations, particularly injection-associated right-sided IE, were retained to demonstrate heterogeneity rather than to determine the principal population estimates. The Italian registry reported by Leone et al. (*n* = 527; 2004–2009) therefore met the principal epidemiological criteria and was included, reporting native-valve involvement of 35.5% aortic, 39.4% mitral, 11.2% tricuspid, and 0.6% pulmonary IE [27]. Evidence was synthesized descriptively rather than through formal quantitative pooling.

No standardized study-level risk-of-bias instrument or formal certainty-of-evidence framework was applied. Methodological characteristics, including study design, sample size, population, and consistency with established pathophysiology, were considered narratively during evidence synthesis; this did not constitute a formal quality appraisal. Findings were integrated thematically to construct a framework linking valve anatomy, hemodynamics, congenital heart disease, microbial factors, and clinical consequences.

## 3. Findings

Across these sources, left-sided IE predominated in population-based cohorts, whereas markedly higher tricuspid involvement was reported in studies enriched for people who inject drugs. Accordingly, Figure 1 above presents descriptive ranges and study-level heterogeneity rather than statistically pooled estimates.

Rather than focusing solely on microbiology or clinical management, this review examines how anatomical architecture, hemodynamic forces, pathological substrates, and structural remodeling collectively determine valve-specific susceptibility across the aortic, mitral, tricuspid, and pulmonary valves. The overall distribution of native-valve infective endocarditis is summarized in Table 1.

### 3.1. Pathobiological Mechanism Underlying Valve Susceptibility in IE

**Figure 2:**
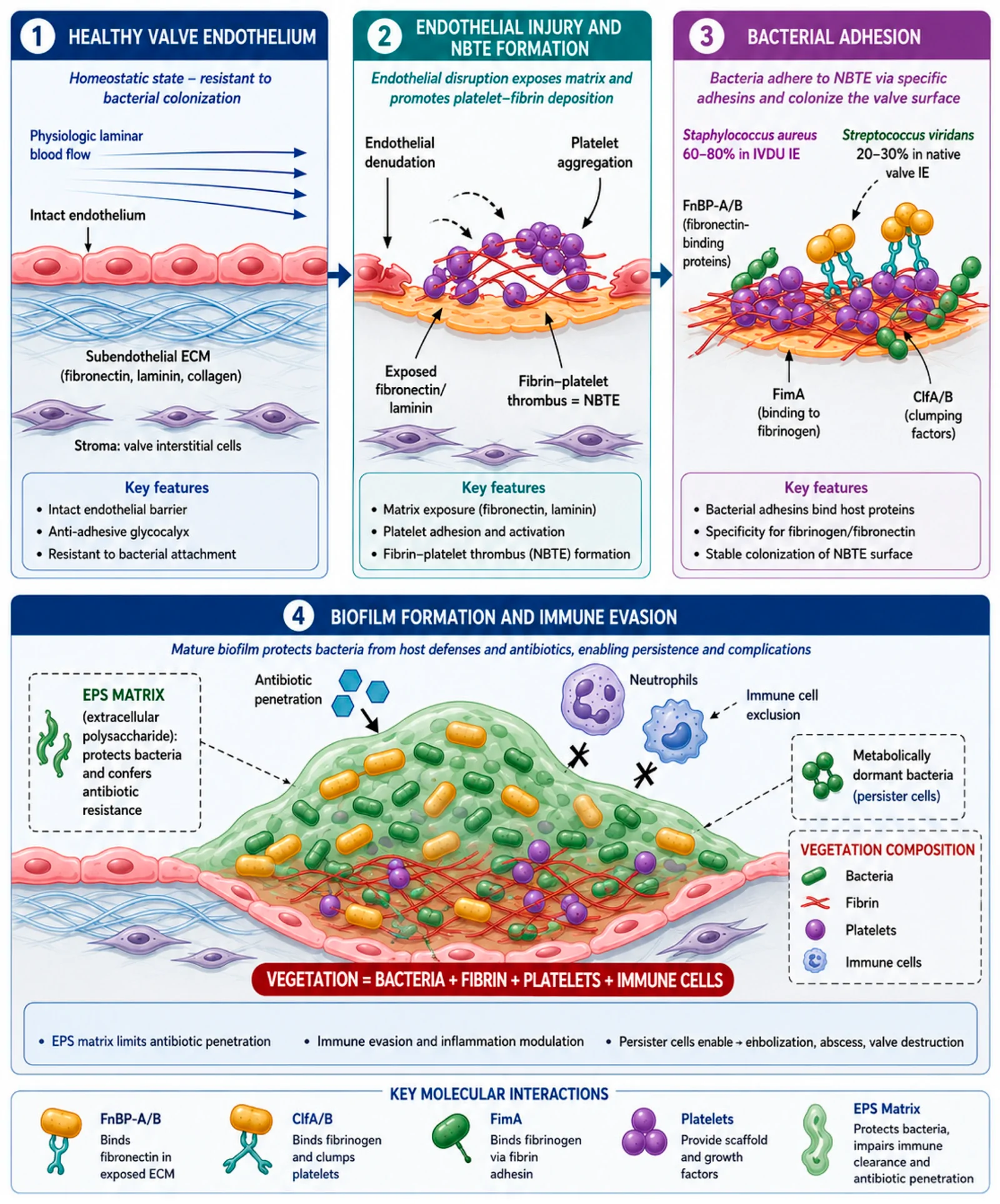
**Molecular pathogenesis of infective endocarditis: from endothelial injury to biofilm-mediated vegetation formation.** (1) Healthy valve endothelium. Under physiological laminar blood flow, the intact valvular endothelium maintains an antithrombotic, anti-adhesive surface that resists bacterial attachment through preservation of endothelial integrity and extracellular matrix homeostasis. (2) Endothelial injury and non-bacterial thrombotic endocarditis (NBTE). Mechanical stress, turbulent flow, or inflammatory injury disrupts the endothelial layer, exposing extracellular matrix proteins, including fibronectin, laminin, and collagen. Platelet activation and fibrin deposition subsequently generate sterile platelet–fibrin thrombi (NBTE), providing the biological substrate for microbial colonization. (3) Bacterial adhesion. Circulating microorganisms attach to the NBTE surface through pathogen-specific adhesins. *Staphylococcus aureus* primarily utilizes fibronectin-binding proteins (FnBP-A/B) and clumping factors (ClfA/B), whereas viridians-group streptococci employ fibrinogen-binding adhesins such as FimA. These molecular interactions facilitate stable bacterial attachment and early vegetation development. (4) Biofilm formation and immune evasion. Following bacterial attachment, microorganisms proliferate within a highly organized platelet–fibrin matrix, forming mature biofilm-like vegetations. The extracellular polymeric substance (EPS) matrix restricts antibiotic penetration, limits immune-cell access, and promotes bacterial persistence, metabolic dormancy (persister cells), and chronic infection. The mature vegetation consists predominantly of bacteria embedded within fibrin, platelets, extracellular matrix, and inflammatory cells, thereby facilitating immune evasion, persistent bacteremia, embolization, and progressive valvular destruction. ECM, extracellular matrix; NBTE, non-bacterial thrombotic endocarditis; FnBP, fibronectin-binding protein; ClfA/B, clumping factor A/B; FimA, fibrinogen-binding adhesin; EPS, extracellular polymeric substance; IVDU, intravenous drug use.

Valve susceptibility in IE reflects a two-step biological process: (i) hemodynamic or inflammatory injury that primes the valvular endocardium, and (ii) microbial exploitation of the resulting platelet–fibrin scaffold and abnormal surfaces [12,13,24,31,32,33]. The convergence of these processes determines not only which valve becomes infected, but also how aggressively infection progresses, particularly in the presence of calcified or prosthetic material [34,35]. The complete molecular pathogenesis cascade is illustrated in Figure 2.

The healthy valvular endothelium is a highly specialized, mechanosensitive biological barrier that actively resists microbial adhesion through preservation of endothelial integrity and maintenance of an antithrombotic, anti-inflammatory phenotype [24,36]. Physiological laminar shear stress maintains endothelial homeostasis by preserving intercellular junctions, suppressing endothelial activation, and maintaining extracellular matrix organization [24,37], thereby preventing platelet adhesion and bacterial colonization [12,13].

This protective phenotype is disrupted by abnormal hemodynamic forces, including turbulent flow, oscillatory shear stress, eccentric regurgitant jets, pressure overload, and repetitive mechanical trauma [12,24,37]. These forces preferentially injure anatomically vulnerable regions, such as leaflet tips, commissures, and flow impingement zones, where endothelial integrity is progressively lost [36,37,38,39]. Classical experimental studies demonstrated that endothelial disruption alone is sufficient to initiate sterile valvular lesions before bacterial colonization occurs [31], a concept subsequently reinforced by modern mechanistic reviews [12,13].

Exposure of subendothelial collagen and extracellular matrix proteins following endothelial disruption rapidly initiates platelet adhesion, fibrin deposition, and sterile thrombus formation [12,13,31]. This lesion, referred to as non-bacterial thrombotic endocarditis (NBTE), constitutes the essential pathological substrate upon which infective endocarditis develops [12,13]. Rather than initiating infection directly, transient bacteremia exploits these pre-existing sterile platelet–fibrin thrombi [12,31].

The platelet–fibrin matrix provides abundant binding sites for bacterial adhesins directed against fibrinogen, fibronectin, and activated platelets [12,13], thereby facilitating microbial attachment and early vegetation formation [13,33]. Similarly, calcified valves [34,40,41,42], prosthetic valves [35,43,44,45,46,47], and cardiovascular implantable electronic devices [48,49] provide abnormal biological surfaces that further promote bacterial adherence and persistence [13,33,35,48,49].

Following bacterial attachment, microorganisms become progressively embedded within the platelet–fibrin scaffold, forming a highly organized biofilm-like vegetation [13,33]. This three-dimensional architecture protects bacteria from hemodynamic shear forces [24], limits penetration of host immune cells [13,33], and restricts antimicrobial diffusion [14,33], thereby promoting persistent infection despite appropriate systemic antibiotic therapy [13,14,33]. Consequently, the platelet–fibrin matrix functions not merely as structural support but as a specialized ecological niche that enhances bacterial survival, immune evasion, and antimicrobial tolerance [13,14,33].

These findings support the contemporary paradigm that infective endocarditis develops through a sequential pathogenic cascade of endothelial injury [12,31], sterile platelet–fibrin thrombus formation [12,13], microbial adhesion [12,13,33], biofilm maturation [13,33], and persistent valvular infection [13,14,33]. This integrated model explains why valve susceptibility is determined by the interaction between local hemodynamic stress [24,37], thrombogenic remodeling [12,13,31], microbial virulence [13,33], and abnormal biological surfaces, including calcified valves [34,40,41,42] and prosthetic valves [35,43,44,45,46,47].

### 3.2. Aortic Valve: The Most Vulnerable Site

The aortic valve represents the most common site of IE, accounting for approximately 35–45% of all native valve infections. Its heightened susceptibility reflects a unique convergence of anatomical, histological, and hemodynamic factors that collectively promote endothelial injury, microbial adherence, tissue destruction, and peri-annular extension. Unlike other cardiac valves, the aortic valve is exposed to the highest-pressure gradients and flow velocities within the circulation, creating a hemodynamic environment that favors endothelial disruption and subsequent bacterial colonization.

Anatomically, the normal aortic valve consists of three semilunar cusps, the right coronary, left coronary, and non-coronary cusps, each composed of three specialized histological layers: the fibrosa, spongiosa, and ventricularis [24]. During systole, the valve is subjected to peak transvalvular velocities of 1.0–1.7 m/s, pressure gradients of 5–10 mmHg, and shear stresses ranging from 10 to 70 dynes/cm^2^, representing the highest hemodynamic burden among the cardiac valves [37]. Comparative hemodynamic parameters across all four valves are summarized in Table 2 [2,7,14,24,28,29,30,37,50,51,52].

A defining anatomical feature of the aortic valve is its intimate continuity with the cardiac fibrous skeleton, including the membranous septum and the mitral–aortic intervalvular fibrosa (MAIVF) [36]. While this fibrous integration provides structural stability, it also creates direct pathways for infection to extend beyond the valve leaflets into surrounding tissues. Consequently, aortic valve IE exhibits the highest rate of peri-annular extension among all valve locations, occurring in approximately 30–40% of cases [53,54].

Underlying structural abnormalities further amplify susceptibility. Bicuspid aortic valve (BAV), affecting approximately 1–2% of the general population [38,39,55], is one of the strongest recognized anatomical risk factors for aortic valve IE and accounts for approximately 24% of aortic infections, conferring a three- to nine-fold increased risk compared with tricuspid aortic valves [56,57]. Similarly, calcific aortic stenosis (AS) significantly increases the risk of IE, affecting approximately 2–7% of adults older than 65 years, with an estimated fivefold increase in infection risk [58]. Structural substrates and relative IE risk across valve types are summarized in Table 3 [6,8,10,11,34,35,38,40,41,42,43,44,45,46,47,52,55,56,57,59,60,61,62,63,64,65,66,67,68,69,70].

Aortic regurgitation (AR) provides an additional hemodynamic mechanism predisposing to infective endocarditis. Chronic regurgitant flow generates high-velocity retrograde jets, typically ranging from 2–5 m/s, which repeatedly strike the ventricular aspect of the anterior mitral leaflet, the left ventricular outflow tract, and adjacent endocardial surfaces [50,55]. Repetitive jet impact produces focal endothelial disruption and characteristic “jet lesions”, exposing subendothelial collagen and extracellular matrix proteins that promote platelet adhesion, fibrin deposition, and non-bacterial thrombotic endocarditis (NBTE), thereby creating a favorable substrate for subsequent bacterial colonization [12,13,24,31]. Unlike aortic stenosis, in which valve susceptibility is primarily driven by calcification and high transvalvular shear stress, AR predisposes to infection predominantly through chronic mechanical injury resulting from eccentric regurgitant flow and repetitive endocardial trauma [24,50,55].

The direction of the regurgitant jet also influences the anatomical distribution of infective lesions. Posteriorly directed jets frequently impinge upon the anterior mitral leaflet, producing secondary “kissing vegetations” or contiguous infection involving both the aortic and mitral valves, whereas centrally directed jets primarily injure the left ventricular aspect of the aortic cusps and adjacent left ventricular outflow tract [47,50,55]. Consequently, severe AR is associated with an increased risk of multivalvular involvement, acute left ventricular volume overload, heart failure, and peri-annular extension when infection becomes established [47,55].

From a clinical perspective, recognition of significant AR should prompt careful echocardiographic assessment of both the aortic valve and adjacent structures, particularly the anterior mitral leaflet, left ventricular outflow tract, and mitral–aortic intervalvular fibrosa, where secondary extension of infection and destructive complications may occur [10,11,16,17,47]. This valve-specific pattern of hemodynamic injury further supports the concept that the direction and magnitude of abnormal flow are important determinants of infective endocarditis susceptibility, lesion distribution, and disease progression, rather than the presence of bacteremia alone [9,12,24].

The clinical consequences of aortic valve IE reflect its unique anatomical relationships. Peri-annular extension frequently results in abscess formation, occurring in approximately 20–40% of cases, most commonly involving the mitral–aortic intervalvular fibrosa [71]. Progressive extension into the membranous septum may disrupt the cardiac conduction system and lead to complete atrioventricular block, reported in 5–10% of patients [71]. Systemic embolization remains a major complication, affecting 20–50% of patients, with cerebral embolization accounting for approximately 65% of all embolic events [72].

### 3.3. Mitral Valve: Conditional Vulnerability

The mitral valve is an equally frequently affected site of IE as the aortic valve, accounting for 35–45% of cases. Unlike the aortic valve, whose susceptibility is largely driven by intrinsic hemodynamic stress, mitral valve infection is predominantly a disease of pre-existing structural vulnerability. In most patients, infection develops upon a substrate altered by degenerative, rheumatic, congenital, or functional abnormalities that disrupt normal endothelial integrity and facilitate microbial colonization.

Anatomically, the mitral valve is a complex apparatus comprising anterior and posterior leaflets, the mitral annulus, chordae tendineae, and papillary muscles [59]. Compared with the aortic valve, the mitral valve is exposed to a lower hemodynamic burden, experiencing peak diastolic flow velocities of 0.6–1.3 m/s, transvalvular pressure gradients of 2–5 mmHg, and shear stresses ranging from 5 to 30 dynes/cm^2^ [73].

MVP represents the most important degenerative substrate predisposing to mitral valve IE. Although MVP affects approximately 2–3% of the general population, it accounts for 20–30% of mitral valve infections and is associated with an estimated eight-fold increase in IE risk [60,61]. Histopathologically, myxomatous degeneration is characterized by proteoglycan accumulation, collagen disorganization, and disruption of normal extracellular matrix architecture, resulting in irregular leaflet surfaces and endothelial dysfunction that facilitate vegetation formation [74].

Mitral regurgitation (MR) provides a complementary hemodynamic mechanism for disease initiation. High-velocity regurgitant jets, often reaching 3–6 m/s in severe MR, repeatedly impact adjacent valvular and endocardial surfaces, producing characteristic “jet lesions” that serve as preferential sites for platelet–fibrin deposition and bacterial adhesion [75].

The anatomical consequences of mitral valve IE differ substantially from those observed in aortic valve disease. Mitral valve infection is associated with lower rates of peri-annular extension (approximately 10–20% of cases) but a markedly greater tendency toward systemic embolization, with embolic events reported in 30–50% of patients and cerebral embolization accounting for approximately 70% of all embolic complications [61,74]. Infection involving the subvalvular apparatus may result in chordal rupture in 10–20% of cases, leading to acute severe mitral regurgitation and urgent surgical intervention [75]. Characteristic echocardiographic features by valve are summarized in Table 4 [10,11,15,16,17,18,19,28,29,30,35,51,53,54,58,71,72,75,76,77,78,79].

In many low- and middle-income countries, rheumatic heart disease remains one of the leading predisposing conditions for mitral valve IE, accounting for approximately 30–50% of reported cases [62]. Progressive rheumatic injury produces leaflet thickening, commissural fusion, chordal shortening, fibrosis, and varying degrees of calcification, resulting in mixed mitral stenosis and regurgitation [62,73]. Beyond increasing susceptibility to infection, these structural abnormalities frequently complicate surgical repair by limiting leaflet mobility and increasing the likelihood of valve replacement rather than reconstruction [55,58]. Consequently, rheumatic mitral valve IE continues to be associated with greater clinical complexity in regions where rheumatic heart disease remains endemic [62].

Mitral annular calcification (MAC) is increasingly recognized as an important degenerative substrate for mitral valve IE, particularly in older adults, among whom its prevalence exceeds 10% [34,40]. However, the independent increase in IE risk attributable to MAC has not been established in population-based longitudinal studies, because the available evidence is derived mainly from descriptive series and selected clinical cohorts [40,41]. In a contemporary cohort of 741 patients evaluated for IE, 51 (7%) had possible or definite IE with moderate-to-severe MAC; mitral-valve involvement was identified in 24 of these 51 patients (47%) [41]. These findings describe the burden of MAC-associated IE within a selected diagnostic cohort and should not be interpreted as the absolute incidence of IE among all patients with MAC. Mechanistically, MAC alters annular geometry, restricts leaflet motion, and produces an irregular calcified surface that may facilitate microbial attachment during bacteremia [34,40]. *Staphylococcus aureus* and coagulase-negative staphylococci are frequently identified in reported MAC-associated IE, consistent with its association with older age, healthcare exposure, and invasive procedures [40,41].

### 3.4. Tricuspid Valve: Low-Pressure Protection with Context-Dependent Risk

The tricuspid valve occupies a unique position within the spectrum of infective endocarditis (IE), demonstrating relatively low intrinsic susceptibility despite continuous exposure to the venous circulation. In contrast to left-sided valves, the tricuspid valve functions within a low-pressure, low-shear hemodynamic environment that confers substantial protection against endothelial injury and vegetation formation. Consequently, tricuspid valve IE accounts for only 5–10% of all cases in the general population [1,2,28,29]. However, this apparent resistance can be rapidly overcome when external factors such as intravenous drug use (IVDU), cardiovascular implantable electronic devices (CIEDs), or congenital heart disease (CHD) alter the native anatomical and hemodynamic environment.

Anatomically, the tricuspid valve consists of three leaflets: the anterior, posterior, and septal leaflets, and possesses the largest annular circumference among the cardiac valves, typically measuring 10–12 cm [62]. Hemodynamically, it is exposed to the lowest transvalvular velocities (0.3–0.7 m/s), pressure gradients (1–3 mmHg), and shear stresses (2–10 dynes/cm^2^) of all four cardiac valves [76]. These physiological characteristics largely explain the relatively low incidence of tricuspid valve IE in structurally normal hearts.

IVDU remains the predominant predisposing condition, accounting for approximately 70–90% of tricuspid valve IE cases [77]. Repeated injection of particulate-contaminated substances causes recurrent endothelial trauma and simultaneously introduces high concentrations of microorganisms directly into the venous circulation. Consequently, the microbiological profile differs substantially from that of left-sided IE, with *Staphylococcus aureus* accounting for 60–80% of infections, polymicrobial pathogens for 10–20%, and fungal organisms for 5–10% [78]. Microbiological distribution according to valve involvement is summarized in Table 5 [2,5,7,10,11,12,13,14,15,26,28,29,30,35,45,46,48,49,60,61,80,81,82,83].

The expanding use of CIEDs, including permanent pacemakers and implantable cardioverter–defibrillators, has introduced another important mechanism of tricuspid valve vulnerability. Transvenous leads traverse the tricuspid valve apparatus and may cause repetitive mechanical trauma, local inflammation, and thrombus formation, thereby increasing the risk of device-related infective endocarditis by approximately two- to fivefold [51,79]. Device infection frequently involves both the intracardiac lead and the adjacent tricuspid valve, often requiring complete device extraction in addition to prolonged antimicrobial therapy [51,55,79].

CHD represents another important anatomical substrate for tricuspid valve infection. Structural abnormalities such as Ebstein anomaly, repaired tetralogy of Fallot, and other complex right-sided congenital lesions are associated with a five- to ten-fold increase in IE risk [30]. Previous surgical reconstruction, prosthetic material, and altered right ventricular geometry further increase susceptibility by creating complex postoperative anatomy that frequently necessitates lifelong clinical surveillance [30,69].

The clinical presentation of tricuspid valve IE differs substantially from that of left-sided disease. Septic pulmonary embolism represents the hallmark complication, occurring in approximately 65–100% of patients [48,84], whereas systemic embolization is uncommon in the absence of an intracardiac shunt. Although overall mortality remains lower than that observed in left-sided IE (10–20% versus 25–40%) [48,84], recurrent infection occurs in approximately 15–30% of patients [48,84], particularly among individuals with persistent intravenous drug use, recurrent healthcare-associated bacteremia, or retained prosthetic material [20,48,84].

In selected patients with large right-sided vegetations, vegetation debulking may provide an alternative strategy for reducing persistent bacteremia and recurrent septic pulmonary embolization while preserving the native valve [20,84]. More recently, the AngioVac percutaneous aspiration system has emerged as a less invasive option for carefully selected patients who are poor surgical candidates or have prohibitive operative risk [20]. Although current evidence remains limited, this technique may reduce vegetation burden and facilitate infection control without conventional open-heart surgery; however, it should complement rather than replace established surgical indications [20,55].

The timing of surgery should be individualized according to hemodynamic status, microbiological response, vegetation size, embolic burden, and the presence of persistent infection [20,55]. Early intervention is generally recommended for uncontrolled sepsis, recurrent septic pulmonary emboli, severe tricuspid regurgitation resulting in right-sided heart failure, or large mobile vegetations despite appropriate antimicrobial therapy [20,55,84]. Following treatment, continued clinical and echocardiographic surveillance is essential because recurrent infection remains an important long-term complication, particularly among patients with ongoing intravenous drug use, CIEDs, or persistent intravascular risk factors [10,11,16,20,48,84].

### 3.5. Pulmonary Valve: The Protected Valve

The pulmonary valve is the least frequently affected native cardiac valve in infective endocarditis (IE), accounting for fewer than 1% of all native valve infections [28,29,64]. This exceptional rarity reflects a unique combination of anatomical isolation, favorable hemodynamics, and reduced exposure to the mechanical stresses that typically initiate endothelial injury and vegetation formation. This exceptional rarity reflects its unique anatomical position within the low-pressure pulmonary circulation, favorable hemodynamics, and limited exposure to the mechanical stresses that typically initiate valvular injury and vegetation formation.

Anatomically, the pulmonary valve consists of three semilunar cusps located at the junction between the right ventricular outflow tract (RVOT) and the pulmonary artery. Compared with all other cardiac valves, it is exposed to the lowest transvalvular pressures and shear forces, experiencing peak flow velocities of only 0.6–0.9 m/s, pressure gradients of 2–5 mmHg, and shear stresses ranging from 1 to 5 dynes/cm^2^ [63]. Consequently, pulmonary valve IE rarely develops in structurally normal hearts and is almost exclusively associated with congenital or surgically modified cardiac anatomy [28,29].

Congenital heart diseases involving the RVOT, particularly tetralogy of Fallot, pulmonary stenosis, and RVOT conduit implantation, represent the principal anatomical substrates for pulmonary valve infection [64,66,67,69]. Repaired tetralogy of Fallot is associated with a ten- to twenty-fold increase in IE risk compared with the general population [85], whereas RVOT conduits and prosthetic pulmonary valve replacements carry reported annual incidences of IE of 3–6% [52]. Given the lifelong survival of patients with repaired congenital heart disease, repeated surgical or transcatheter interventions further increase cumulative exposure to prosthetic material and healthcare-associated bacteremia, thereby contributing to the growing incidence of pulmonary valve IE [20,52,69].

Among congenital lesions, pulmonary stenosis represents the most important hemodynamic risk factor. Persistent RVOT obstruction generates high-velocity systolic jets that repeatedly impact the pulmonary valve and adjacent endocardium, producing characteristic jet lesions that predispose to infection [52,64,66]. The risk is particularly pronounced in patients with residual obstruction following congenital heart disease repair or in those with prosthetic RVOT conduits, where abnormal postoperative anatomy further increases susceptibility to IE [66,67,69].

In contrast, pulmonary regurgitation most commonly develops following repair of tetralogy of Fallot, pulmonary valve replacement, or RVOT reconstruction [69,85]. Although isolated pulmonary regurgitation is not considered an independent predisposing factor for IE, severe regurgitation frequently coexists with homograft, bioprosthetic valves, or transcatheter pulmonary valve prostheses, all of which are recognized substrates for infection [52,67,69,85]. Progressive right ventricular volume overload and RVOT remodeling secondary to chronic pulmonary regurgitation also influence the timing of pulmonary valve intervention before irreversible right ventricular dysfunction develops [23,69].

The increasing adoption of transcatheter pulmonary valve replacement (TPVR) has expanded therapeutic options for dysfunctional RVOT conduits and bioprosthetic pulmonary valves, particularly in adults with repaired congenital heart disease [69]. Despite favorable hemodynamic outcomes and avoidance of repeat sternotomy, IE has emerged as one of the most important late complications following TPVR, necessitating continued clinical vigilance and lifelong surveillance [20,69]. Likewise, homograft and valve conduit infections remain important causes of pulmonary valve IE because progressive conduit degeneration and repeated interventions may predispose to prosthetic infection and peri-conduit extension [19,52,69]. Patients undergoing the Ross procedure, in which the native pulmonary valve is transferred to the aortic position and the RVOT is reconstructed with a pulmonary homograft, require particular attention because infection may involve the reconstructed outflow tract, creating unique diagnostic and surgical challenges [55,69].

The Ross procedure provides an illustrative clinical model for considering the relative contributions of intrinsic valve biology and hemodynamic environment. In this operation, the native pulmonary valve is transferred to the systemic aortic position and becomes exposed to higher pressure and altered flow conditions. Gamez et al. reported longitudinal follow-up of 92 patients who underwent the Ross procedure, among whom four developed infective endocarditis involving the pulmonary homograft [86]. Although this observation does not establish causality, it raises the possibility that pulmonary valve tissue exposed to systemic hemodynamic conditions following the Ross procedure may be more susceptible to infective endocarditis than in its native right-sided position. It therefore suggests that both intrinsic tissue characteristics and hemodynamic stress should be considered when interpreting valve-specific IE risk.

The clinical manifestations of pulmonary valve IE are generally associated with the most favorable prognosis among all cardiac valves, with reported mortality rates of only 5–15% [64]. Nevertheless, acute pulmonary regurgitation may precipitate severe right ventricular volume overload and right-sided heart failure in approximately 30–50% of affected patients [64]. Management is guided by the severity of valvular dysfunction, hemodynamic compromise, persistent infection, embolic complications, and prosthetic valve involvement. Early surgical intervention is recommended for uncontrolled infection, extensive pulmonary valve destruction, RVOT conduit infection, or progressive right ventricular failure, whereas valve-preserving procedures should be considered whenever anatomically feasible [20,52,55,69].

### 3.6. Patient Specific Modifiers of Valve-Specific Susceptibility

Although intrinsic valve anatomy establishes the baseline biological susceptibility to infective endocarditis (IE), the clinical expression of disease is substantially influenced by patient-specific modifiers that alter endothelial integrity, hemodynamic conditions, microbial exposure, prosthetic material, or host immune defense [9,12,13,24,31]. Rather than replacing the influence of valve anatomy, these factors interact with valve-specific structural characteristics to amplify or redirect the underlying biological susceptibility, thereby modifying the anatomical distribution, microbiological profile, disease severity, and clinical outcome of IE.

**Figure 3:**
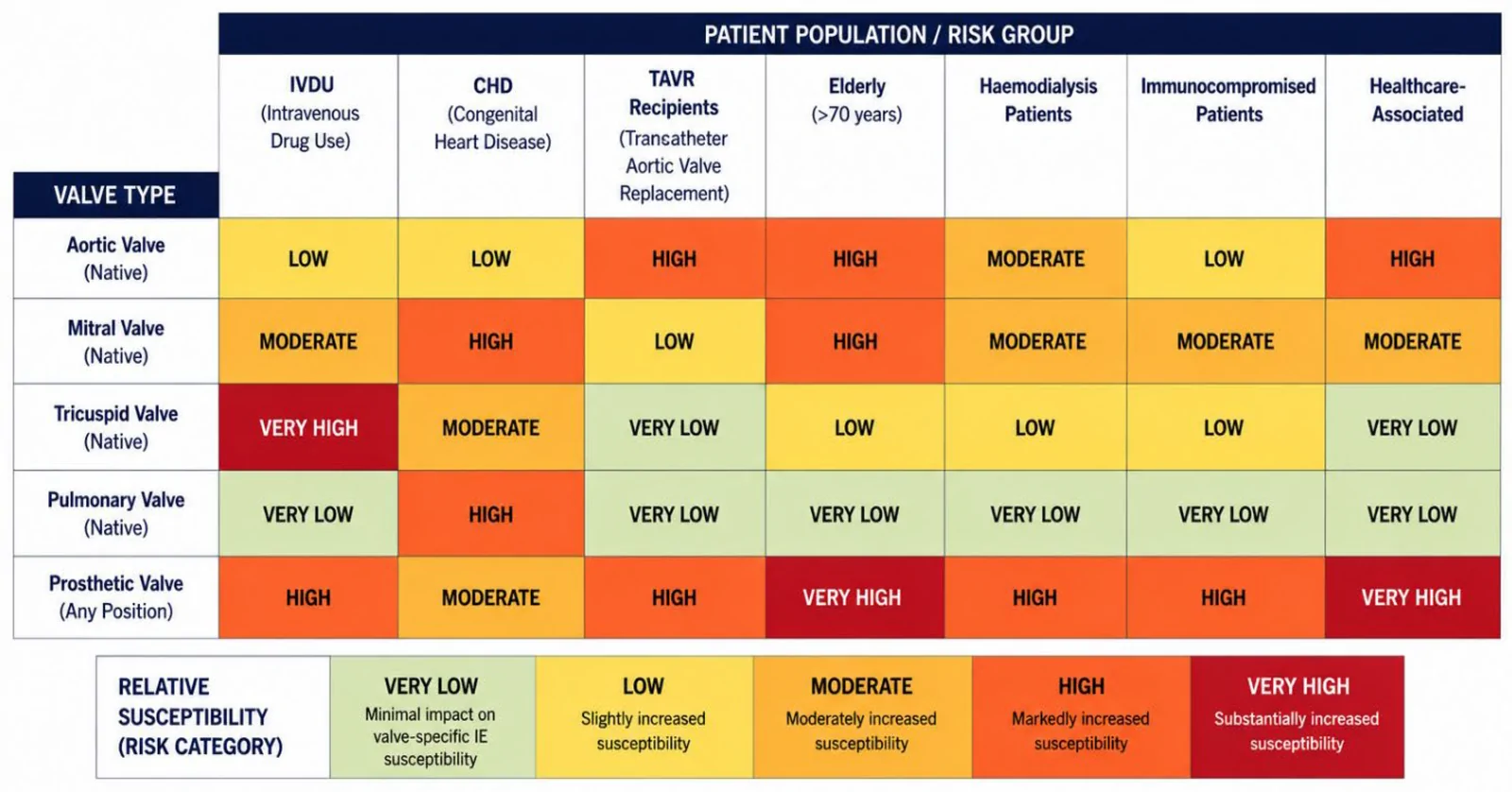
**Patient-specific modifiers of valve-specific susceptibility to infective endocarditis.** Qualitative heatmap demonstrating how major patient-specific clinical conditions modify the baseline valve-specific susceptibility to infective endocarditis. Color intensity represents the proportion of IE episodes within a specified patient population involving the indicated valve: very low, <5%; low, 5–14%; moderate, 15–29%; high, 30–49%; and very high, ≥50%.

Figure 3 illustrates this concept by demonstrating how major patient populations influence the relative susceptibility of different cardiac valves. The qualitative heatmap should be interpreted as a conceptual synthesis of the current literature rather than a quantitative estimate of disease incidence. It highlights the interaction between intrinsic valve biology and patient-specific clinical conditions that collectively determine the observed patterns of valve involvement in contemporary IE [2,8,9,10,14,24,28,29,30,35,45,46,47,48,49,55,64,65,66,67,68,69,80,81,82].

Among these modifiers, intravenous drug use (IVDU) preferentially increases susceptibility of the tricuspid valve because repeated venous inoculation of microorganisms and particulate contaminants produces direct endothelial injury within the low-pressure right-sided circulation [48,77,78,84]. Conversely, congenital heart disease (CHD) predominantly affects the pulmonary and right-sided valves through abnormal intracardiac hemodynamic, residual shunts, prosthetic conduits, and previous surgical reconstruction [30,52,64,65,66,67,68,69,85]. Transcatheter aortic valve replacement (TAVR), prosthetic valves, and cardiovascular implantable electronic devices (CIEDs) create artificial biological surfaces that facilitate platelet–fibrin deposition, bacterial adhesion, and biofilm formation, thereby increasing susceptibility to prosthetic valve endocarditis and device-related infection [15,18,20,35,49,51,79].

Systemic conditions further modify valve susceptibility through mechanisms distinct from structural heart disease. Advanced age, hemodialysis, immunocompromised states, and healthcare-associated exposure increase the frequency of bloodstream infection, promote colonization by multidrug-resistant organisms, and impair host defense, thereby amplifying the risk of infective endocarditis, particularly in patients with pre-existing degenerative valve disease or prosthetic material [2,15,18,20,35,49]. These factors explain the evolving epidemiology of IE, in which healthcare-associated infection and prosthetic valve endocarditis now account for an increasing proportion of cases despite the declining prevalence of rheumatic heart disease [2,3,7,8]. The principal biological mechanisms through which these patient-specific factors modify valve susceptibility are summarized in Table 6 [2,9,12,13,15,16,17,18,19,20,21,22,23,24,28,29,30,31,35,48,49,51,52,63,64,65,66,67,68,69,77,78,79,84,85,87]. Diabetes mellitus is also associated with increased susceptibility to IE through impaired host defense and greater exposure to bloodstream infection; however, no consistent valve-specific predominance has been established [87].

### 3.7. Prosthetic Valves: Artificial Substrates and Altered Anatomical Risk

**Figure 4:**
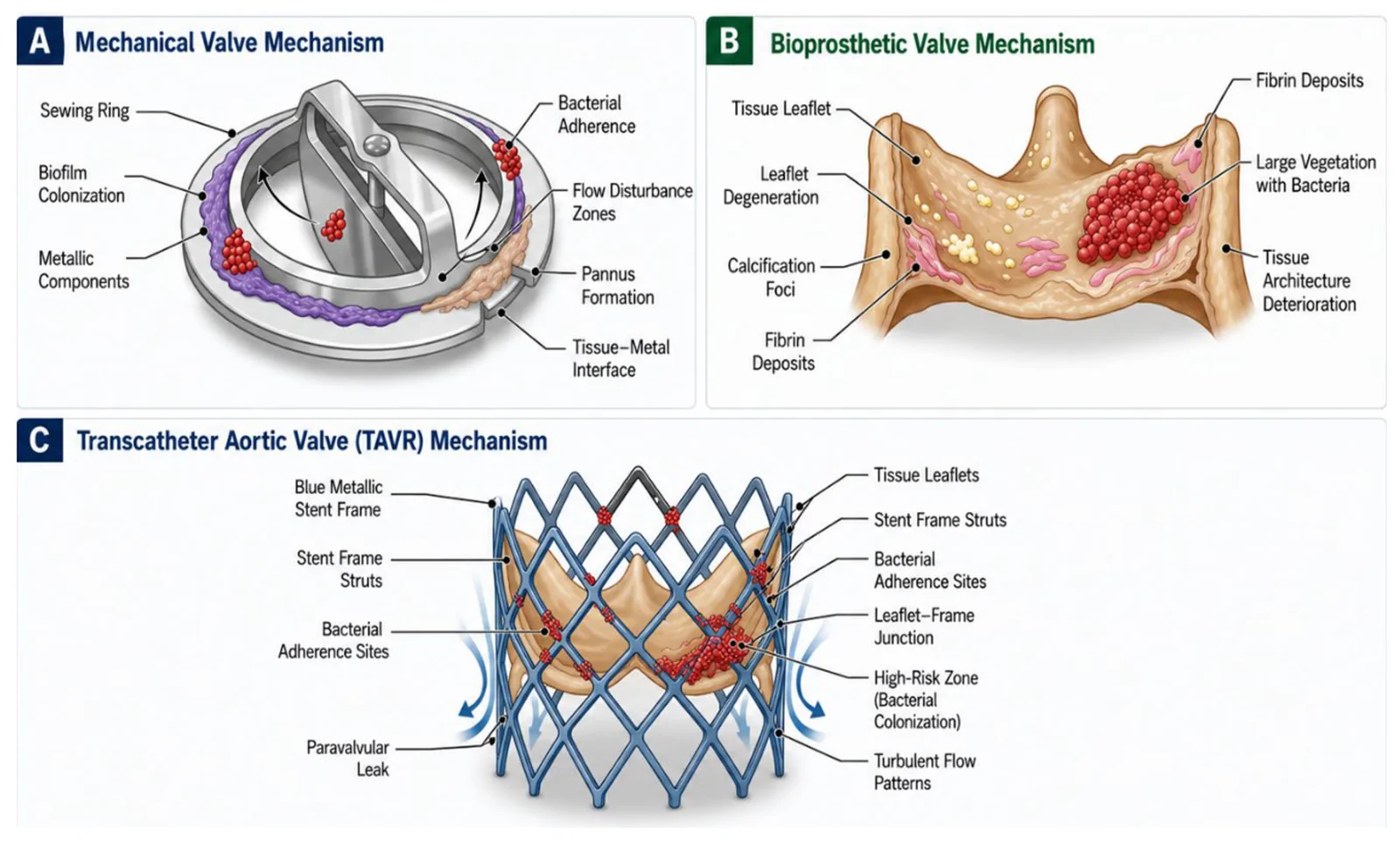
**Mechanisms of Prosthetic Valve Infective Endocarditis Across Different Valve Types.** Schematic illustration of the structural mechanisms predisposing different prosthetic valve types to infective endocarditis (IE). (**A**) Mechanical prosthetic valves are susceptible to bacterial adherence primarily at the sewing ring and tissue–metal interface, where biofilm formation, pannus development, and local flow disturbances facilitate microbial colonization. (**B**) Bioprosthetic valves develop IE through progressive leaflet degeneration, calcification, fibrin deposition, and extracellular matrix deterioration, providing a favorable substrate for vegetation formation and bacterial persistence. (**C**) Transcatheter aortic valve replacement (TAVR) prostheses exhibit unique sites of bacterial attachment along the metallic stent frame and leaflet–frame junctions, where turbulent flow, paravalvular leak, and exposed metallic struts promote biofilm formation and microbial colonization.

Prosthetic valve implantation fundamentally alters the anatomical and biological environment of the native valve, creating an artificial substrate that is uniquely susceptible to IE. Unlike native valve infection, where susceptibility is largely determined by intrinsic anatomy and hemodynamics, prosthetic valve endocarditis (PVE) arises from the interaction between foreign material, altered flow patterns, host immune responses, and microbial biofilm formation. The spectrum of prosthetic valve types and their IE risk profiles is illustrated in Figure 4 and summarized in Table 7 [14,35,43,44,45,46,47,80,81,82,88].

The anatomical distribution of PVE broadly parallels that of native-valve IE, with the aortic prosthesis more frequently involved than the mitral prosthesis in reported clinical series [88]. However, the observed distribution is influenced by the relative number of valves implanted at each position and by differences in study populations; therefore, these proportions should not be interpreted as intrinsic position-specific incidence rates. Across historical surgical-valve cohorts, the cumulative incidence of PVE has been reported at approximately 1.4–3.1% at 1 year and 3–5.7% at 5 years [88]. Although mechanical prostheses may carry a greater risk during the early postoperative period and bioprosthetic susceptibility may increase later as structural degeneration develops, cumulative PVE occurrence at 5 years appears broadly similar between mechanical and bioprosthetic valves [88].

Mechanical prosthetic valves are associated with an annual IE incidence of approximately 0.3–1.2% [80]. Early prosthetic valve endocarditis (within 60 days of implantation) is predominantly related to perioperative contamination, with *Staphylococcus aureus* and coagulase-negative staphylococci as the principal causative organisms [81]. Owing to the intimate relationship between the prosthesis, annulus, and surrounding cardiac structures, early PVE is characterized by aggressive local invasion, with peri-annular extension occurring in approximately 40–60% of cases and reported mortality rates of 20–40% [81].

Bioprosthetic valves are similarly vulnerable to infection, with annual IE incidences ranging from 0.5–1.5% [82]. Structural valve degeneration progressively increases susceptibility over time, typically becoming apparent after 7–10 years.

The emergence of transcatheter aortic valve replacement (TAVR) has introduced a distinct and increasingly important form of prosthetic valve infection. Reported incidences of TAVR-associated IE range from 0.3–3.1% per year [14,35]. Several device-specific anatomical factors have been implicated in susceptibility, including paravalvular regurgitation reported in approximately 22% of TAVR-associated IE cases as moderate-to-severe residual aortic regurgitation [45], residual calcified native valve tissue, and disruption of normal valvular architecture [35,43]. Paravalvular regurgitation creates turbulent flow and endothelial injury at the prosthesis–tissue interface, providing a nidus for microbial colonization [44]. Enterococcus species account for approximately 25–35% of TAVR-related infections, and clinical outcomes remain poor, with mortality rates ranging from 40–60% [44].

## 4. Limitations

This review has several limitations related to its search strategy. First, the electronic searches were based primarily on iteratively developed free-text terms rather than a validated combination of Medical Subject Headings (MeSH), Emtree terms and database-specific controlled vocabulary. The term ‘risk’ was not explicitly included. Consequently, relevant studies indexed predominantly under risk, predictors, incidence, predisposition or related epidemiological terminology may not have been retrieved. Conversely, ‘susceptibility’ is a conceptual term and is not a uniform or standardized synonym for epidemiological risk. Its use may have preferentially identified mechanistic and qualitative publications rather than studies reporting directly measured incidence or relative-risk estimates, thereby introducing search and selection bias.

Second, this was a narrative rather than a systematic review. Although literature identification and study selection were structured, the review did not incorporate all safeguards expected of a systematic review, including a prospectively registered protocol, a fully exhaustive controlled-vocabulary search, formal study-level risk-of-bias assessment and certainty-of-evidence grading. The evidence base may therefore be less complete and less reproducible, and the relative weighting of individual studies may be influenced by author judgement. In addition, heterogeneity in study populations, definitions, valve types, follow-up intervals and outcome reporting precluded quantitative pooling. Numerical ranges presented in the tables and figures should therefore be interpreted as descriptive syntheses of heterogeneous literature rather than pooled or validated risk estimates. These limitations mean that the proposed valve-specific framework should be regarded as an evidence-informed conceptual synthesis that requires confirmation in prospective and systematically designed studies.

## 5. Conclusions

Infective endocarditis is fundamentally a valve-specific disease, in which susceptibility is determined by the complex interaction between anatomical architecture, hemodynamic forces, structural remodeling, microbial virulence, and patient-specific clinical factors. Rather than affecting cardiac valves uniformly, IE develops preferentially in valves possessing distinct structural and biomechanical characteristics that favor endothelial injury, thrombus formation, microbial adhesion, and vegetation maturation. Throughout this review, the aortic, mitral, tricuspid, and pulmonary valves have each demonstrated unique anatomical substrates, pathological mechanisms, imaging characteristics, and clinical behaviors that collectively explain their markedly different susceptibilities to infection [9,10,12,13,24].

Advances in multimodality imaging, structural heart interventions, and cardiac surgery have further highlighted the importance of understanding valve-specific anatomy in the diagnosis and management of IE [10,11,15,16,17,18,19,20,21,22,23,55]. Recognition of these anatomical and hemodynamic differences not only improves diagnostic accuracy but also informs risk stratification, surgical planning, prosthetic valve selection, and postoperative surveillance, particularly in patients with congenital heart disease, prosthetic valves, cardiovascular implantable electronic devices, and other complex structural substrates [15,16,17,18,19,20,21,22,23,35,48,49,55,69].

## Figures and Tables

**Table 1:** Published studies informing Figure 1, arranged chronologically by publication year to demonstrate temporal and quantitative heterogeneity in reported native-valve infective endocarditis distribution.

Author (Year)	Period	Region	*n*	Aortic (%)	Mitral (%)	Tricuspid (%)	Pulmonary (%)
Hecht & Berger 1992 [28]	1981–1990	USA	102	~0	~0	~95	~5
Frontera & Gradon 2000 [29]	Review	USA	NR	~20	~15	~65	~5
Miro et al. 2002 [30]	Review	Spain	~200	~12	~12	~71	~5
Hoen et al. 2002 [5]	1999	France	390	~43	~39	~8	~1
Murdoch et al. 2009 [2]	2000–2005	International	2781	~41	~38	~10	~1
Leone et al. 2012 [27]	2004–2009	Italy	527	35.5	39.4	11.2	0.6
Hoen & Duval 2013 [14]	Review	International	NR	~41	~38	~10	<1
Slipczuk et al. 2013 [7]	1950–2012	International	NR	~41	~34	~7	~2

NR = no record.

**Table 2:** Valve-Specific Hemodynamic Parameters and IE Risk Correlation.

Valve	Peak Velocity (m/s)	Pressure Gradient (mmHg)	Shear Environment	Native IE Frequency	Relative IE Risk
Aortic	1.0–1.7	5–10	Spatially and temporally heterogeneous; experimental aortic-surface values are study-specific and approximately 0–20 dynes/cm^2^	35–45%	Highest
Mitral	0.6–1.3	2–5	Complex phasic and oscillatory leaflet shear; altered by regurgitant jets	35–45%	High (with substrate)
Tricuspid	0.3–0.7	1–3	Lower-pressure, respiration-dependent flow; modified by regurgitation, devices and abnormal anatomy	5–10% (30–50% IVDU)	Low–High (context-dependent)
Pulmonary	0.6–0.9	2–5	Low-pressure pulsatile outflow; modified by congenital lesions and conduits	<1%	Lowest

IVDU = intravenous drug users. Risk estimates represent native valve IE in the general population. Hemodynamic values represent the normal physiological range.

**Table 3:** Structural Substrates and Relative IE Risk by Valve Type.

Structural Substrate	Valve Affected	Epidemiological Context	Association with IE	Mechanism
BAV	Aortic	Present in approximately 1–2% of the population	Substantially increased IE susceptibility	Asymmetric flow, raphe endothelial disruption
Calcific aortic stenosis	Aortic	Prevalence increases markedly with age	Increased susceptibility; magnitude not consistently quantified	Surface irregularity, turbulent flow >4 m/s
MVP	Mitral	Present in approximately 2–3% of the population	Increased susceptibility, particularly with significant mitral regurgitation or flail leaflet	Myxomatous degeneration, jet lesions
MAC	Mitral	>10% in older adults	Recognized IE substrate; independent relative risk not quantified	Irregular calcified annular surface, degenerative valve disease
Rheumatic heart disease	Mitral	Prevalence varies substantially by geographical region	Recognized predisposing condition; magnitude varies by population	Leaflet thickening, commissural fusion
Ebstein anomaly	Tricuspid	Rare congenital abnormality	Increased susceptibility recognized but poorly quantified	Abnormal leaflet morphology, turbulent flow
Repaired tetralogy of Fallot	Pulmonary	Risk depends on residual lesions, conduits and pulmonary-valve replacement	Markedly increased after pulmonary-valve or conduit intervention	RVOT reconstruction, residual gradients
Surgical prosthetic valve	Any	Risk varies by valve position, prosthesis type, postoperative timing and follow-up duration	Established high-risk substrate for IE	Non-endothelialized surface, biofilm
TAVR device	Aortic	Reported incidence approximately 0.3–2.0 per 100 person-years	Established risk of prosthetic aortic-valve IE	Paravalvular leak, residual native tissue

RVOT = right ventricular outflow tract; TAVR = transcatheter aortic valve replacement, BAV = bicuspid aortic valve, MVP = mitral valve prolapse, MAC = mitral annular calcification.

**Table 4:** Echocardiographic Features and Anatomical Complications by Valve.

Feature or Complication	Aortic IE	Mitral IE	Tricuspid IE	Pulmonary IE	Prosthetic-Valve IE
Vegetation location	Ventricular aspect; cusp tips	Atrial aspect; leaflet edges	Atrial aspect; leaflet tips	Ventricular aspect of pulmonary cusps	Sewing ring, prosthesis–tissue interface or prosthetic leaflet
Reported vegetation size	5–15 mm ^b^	8–20 mm ^b^	10–30 mm ^b^	5–15 mm ^b^	Variable
Periannular extension	30–40% ^b^	10–20% ^b^	<5% ^b^	Rare/not reliably quantified	55% ^a^
Abscess formation	20–40% ^b^	10–15% ^b^	<5% ^b^	Rare/not reliably quantified	40–60% ^b^
Valvular perforation or tear	10–20% ^b^	10–20% ^b^	5–10% ^b^	5–10% ^b^	15–25% ^b^
Intracardiac fistula	Predominantly aortic; 1.8% across native IE ^a^	Rare	0.4% in drug-associated IE ^a^	Rare	3.5% ^a^
Conduction disturbance/AV block	5–10% ^b^	2–5% ^b^	<2% ^b^	Rare/not reliably quantified	10–20% ^b^
Systemic embolism	20–50% ^b^	30–50% ^b^	Uncommon	Rare	20–40% ^b^
Septic pulmonary embolism	Rare	Rare	Common; reported range varies ^b^	Rare	Rare unless right-sided
TTE sensitivity	40–60% for native-valve IE ^a^	40–60% for native-valve IE ^a^	Not independently established	Not independently established	Approximately 17–40% ^a^
TEE sensitivity	90–100% for native-valve IE ^a^	90–100% for native-valve IE ^a^	Not independently established	Not independently established	Approximately 82–96% ^a^

Values marked ^a^ were directly reported in the cited studies. Values marked ^b^ represent approximate narrative ranges synthesised from heterogeneous observational studies, surgical series, guidelines and reviews and should not be interpreted as pooled estimates. TTE = transthoracic echocardiography, TEE = transesophageal echocardiography.

**Table 5:** Microbiology of IE by Valve Site and Clinical Context.

Pathogen	Left-Sided Native IE: Aortic/Mitral (%)	Right-Sided/Tricuspid IE (%)	Prosthetic IE (%)	Key Clinical Context
*Staphylococcus aureus*	25–35	60–80	20–30 (early)	IVDU, healthcare-associated, community
Coagulase-negative staphylococci	10–15	5–10	30–40 (early)	Prosthetic valves, healthcare-associated
*Streptococcus viridians* group	25–40	5–10	15–25 (late)	Dental procedures, community-acquired
*Enterococcus* spp.	10–15	2–5	10–15; 25–35 (TAVR)	Elderly, GI/GU procedures, TAVR
*Streptococcus bovis*/*gallolyticus*	5–10	<2	5–10 (late)	Colorectal pathology, elderly
HACEK organisms	2–5	<2	2–5	Subacute presentation, dental source
Fungi (Candida/Aspergillus)	2–5	5–10	5–10	IVDU, immunocompromised, prolonged ICU
Polymicrobial	2–5	10–20	5–10	IVDU, healthcare-associated
Culture-negative	5–10	5–10	10–15	Prior antibiotics, fastidious organisms

IVDU = intravenous drug users; GI = gastrointestinal; GU = genitourinary; HACEK = *Haemophilus*, *Aggregatibacter*, *Cardiobacterium*, *Eikenella*, *Kingella*; ICU = intensive care unit; TAVR = transcatheter aortic valve replacement.

**Table 6:** Biological mechanisms through which patient-specific factors modify valve-specific susceptibility to infective endocarditis.

Patient-Specific Modifier	Biological Mechanism	Valves Predominantly Affected	Major Pathogens	Clinical Implication
IVDU	Venous inoculation + endothelial injury	Tricuspid	*S. aureus*	Septic pulmonary emboli
CHD	Abnormal hemodynamic	Pulmonary	Streptococci	Lifelong surveillance
TAVR	Prosthetic surface	Aortic	Enterococcus	PET/CT
Hemodialysis	Repeated bacteremia	Prosthetic	*S. aureus*	Early TEE
Immunocompromised	Impaired immunity	Any	Fungi, Gram-negative	Early diagnosis
Healthcare- associated	Resistant organisms	Prosthetic	MRSA, Enterococcus	MDT
Diabetes mellitus	Impaired host defense and increased bacteremia exposure	Any	No diabetes-specific distribution established	Heightened clinical vigilance

PET = positron emission tomography, CT = computed tomography, MRSA = methicillin-resistant *Staphylococcus aureus*, MDT = multidisciplinary team.

**Table 7:** Prosthetic Valve Types: Anatomical Characteristics and IE Risk Profiles.

Characteristic	Mechanical Valve	Bioprosthetic Valve	TAVR Device
Annual IE incidence	0.3–1.2%	0.5–1.5%	0.3–3.1%
Peak risk period	First 60 days (early PVE)	First 60 days + after 7–10 yrs	First year post-implant
Surface characteristics	Non-endothelialized metallic/pyrolytic carbon	Partial endothelialisation degenerates over time	Residual native leaflets + stent frame
Predominant early pathogens	*S. aureus*, CoNS	*S. aureus*, CoNS	*S. aureus*, *Enterococcus* spp.
Predominant late pathogens	Streptococci, enterococci	Streptococci, enterococci	*Enterococcus* spp. (25–35%)
Periannular extension rate	40–60%	30–50%	30–50%
Paravalvular regurgitation	Uncommon (<5%)	Uncommon (<5%)	30–50% (mild–moderate)
Surgical re-intervention rate	High	Moderate	Very high (40–60% mortality)
Mortality (IE episode)	20–40%	20–40%	40–60%
Key anatomical risk factor	Prosthesis–annulus interface	Leaflet calcification/degeneration	Paravalvular gap, native valve remnant

TAVR = transcatheter aortic valve replacement; CoNS = coagulase-negative staphylococci; PVE = prosthetic valve endocarditis; RVOT = right ventricular outflow tract; IE = infective endocarditis.

## Data Availability

No new data were created or analyzed in this study. Data sharing is not applicable to this article.

## Supplementary Materials

The following supporting information can be downloaded at: https://www.mdpi.com/article/10.3390/jcm15166281/s1, Figure S1. PRISMA 2020 flow diagram illustrating the literature search and study selection process. Records were identified from PubMed/MEDLINE (*n* = 312), Embase (*n* = 287), Scopus (*n* = 198), and Google Scholar (*n* = 453); 85 studies were ultimately included in the narrative synthesis.
