# Supplementary material for: Valve-Specific Anatomy and Structural Determinants of Susceptibility to Infective Endocarditis: A Review
Source: J Clin Med. 2026 Aug 13;15(16):6281. doi: 10.3390/jcm15166281 (PMC13513480; doi:10.3390/jcm15166281)
Supplement: Supplementary file 1 [file jcm-15-06281-s001.zip › jcm-4377845-supplementary.pdf]

Journal of
Clinical Medicine

MDPI

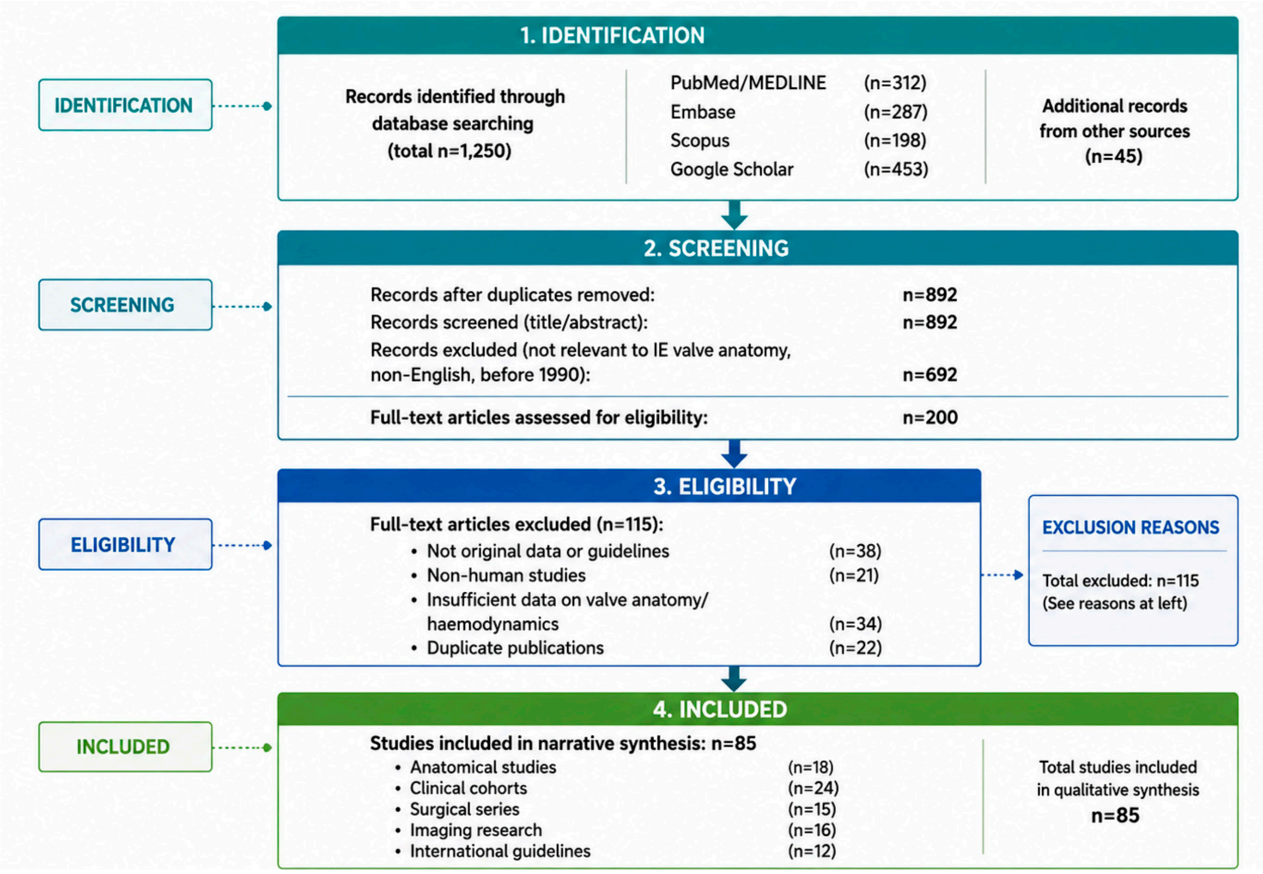

Figure S1. PRISMA 2020 flow diagram illustrating the literature search and study selection process. Records were identified from PubMed/MEDLINE (n = 312), Embase (n = 287), Scopus (n = 198), and Google Scholar (n = 453); 85 studies were ultimately included in the narrative synthesis.
